# Cytotoxic Lesion of the Corpus Callosum Related to Migraine With Aura Triggered by In Vitro Fertilization and Embryo Transfer: A Case Report

**DOI:** 10.7759/cureus.86200

**Published:** 2025-06-17

**Authors:** Yasemin Akinci, Gregory Anash

**Affiliations:** 1 Department of Neurology, Istanbul University-Cerrahpasa, Cerrahpasa Faculty of Medicine, Istanbul, TUR; 2 Department of Neurology, JFK University Medical Center, Hackensack Meridian Health, Edison, USA; 3 Department of Child Neurology, Jersey Shore University Medical Center, Hackensack Meridian Health, Neptune, USA

**Keywords:** corpus callosum, cytotoxic lesion of the corpus callosum, in vitro fertilization, migraine, migraine with aura

## Abstract

Cytotoxic lesions of the corpus callosum (CLOCCs) have been linked to a wide range of clinical conditions and, though rarely reported, have also been observed in patients with migraine. While their exact pathophysiology is not fully understood, CLOCCs are thought to result from complex inflammatory cascades that trigger cytokine storms and oxidative stress. Most reported cases are reversible and carry a favorable short-term clinical prognosis, though long-term outcomes remain unclear. In vitro fertilization and embryo transfer (IVF-ET), which involves pronounced hormonal fluctuations, may contribute to varying degrees of headache, particularly in individuals with a history of migraine. We present the case of a 26-year-old woman who developed migraine with aura episodes after initiating IVF-ET treatment. Her most severe migraine episode occurred following IVF-ET failure, which likely led to a sharp drop in estrogen levels, and was associated with the development of a cytotoxic lesion in the splenium of the corpus callosum. Follow-up brain magnetic resonance imaging at six months showed near-complete lesion resolution, and the patient did not show any signs of permanent neurological sequelae during this period. To date, and to our knowledge, this is the first reported case in the literature of a CLOCC triggered by IVF-ET-associated migraine.

## Introduction

Cytotoxic lesions of the corpus callosum (CLOCCs) have been reported as secondary to a diverse range of clinical conditions, including viral and bacterial infections, epilepsy, drug and toxin exposure, subarachnoid hemorrhage, cerebral venous thrombosis (CVT), autoimmune disorders, trauma, neoplasia, metabolic disturbances, alcoholism, eclampsia, certain vaccines, high-altitude sickness, vitamin B12 deficiency, deep brain stimulation, and Parkinsonism [1,2]. A limited number of pediatric and adult cases in the literature have linked CLOCCs to migraine, both with and without aura [3,4].

These lesions typically exhibit features of cytotoxic edema on cranial magnetic resonance imaging (MRI), including hyperintensity on diffusion-weighted imaging (DWI) with corresponding hypointensity on apparent diffusion coefficient (ADC) mapping, hyperintensity on T2-weighted and fluid-attenuated inversion recovery (FLAIR) sequences, and hypointensity on T1-weighted images without gadolinium enhancement. Computed tomography (CT) may reveal hypoattenuating lesions. Although CLOCCs usually present as small, central, ovoid, or round lesions in the splenium, they can extend into the adjacent white matter and anterior corpus callosum (CC) [5].

The clinical manifestations of CLOCCs reflect those of the underlying condition. In cases associated with migraine, they are generally considered benign in the short term. Radiological findings typically resolve within weeks to months, though not always completely. In vitro fertilization and embryo transfer (IVF-ET) therapy has been associated with headaches, particularly in individuals with a history of migraine, as reported in a retrospective study [6]. However, to date, no study has investigated the precise mechanism of migraine related to IVF-ET or its short- and long-term complications.

Here, we present a case of a patient who developed migraine with aura (MwA) episodes and an associated CLOCC following IVF-ET treatment. This case highlights the importance of monitoring and managing headaches in women undergoing IVF-ET to improve their quality of life and reduce the risk of migraine-related complications. 

## Case presentation

A 26-year-old woman with a history of headaches, polycystic ovary syndrome, insulin resistance, and hypothyroidism presented with a one-day history of severe headaches. The episode was preceded by blurred vision and zigzag lines in the right visual field, lasting approximately 20 minutes. The headache was throbbing in nature, rated nine out of 10 in severity, localized to the left side of the head with the most intense pain behind the left eye, and accompanied by nausea and photophobia. While the aura and headache characteristics resembled her usual episodes, the severity was unprecedented. She was unable to sleep due to the pain, an unusual occurrence for her, which prompted her to seek emergency medical care.

Her headache episodes began 1.5 years earlier, following the initiation of IVF-ET protocols, and fulfilled the diagnostic criteria for MwA based on the International Classification of Headache Disorders, 3rd edition (ICHD-3) [7]. Over the preceding 1.5 years, she underwent three cycles of clomiphene citrate and three cycles of letrozole for ovarian stimulation and follicular response. She had also been prescribed various medications as part of her IVF-ET treatment regimen, including oral estradiol hemihydrate (4 mg/day), oral azithromycin (500 mg/day), oral aspirin (100 mg/day), vaginal and intramuscular progesterone (50 mg/day), subcutaneous menotropin (150 IU/day), subcutaneous cetrorelix acetate (0.25 mg/day), subcutaneous follitropin alfa (450 IU/day), and subcutaneous enoxaparin (40 mg/day).

Following the initiation of IVF-ET, she began experiencing migraine attacks with visual aura two to three times per month. These episodes were managed with over-the-counter dexketoprofen trometamol and paracetamol, which provided adequate relief. As there were no other significant lifestyle changes during this period, she attributed the headaches to fertility treatments and did not seek medical evaluation.

A blood pregnancy test performed 10 days prior to symptom onset was negative, and she had discontinued all IVF-ET treatments following this result. At the time of presentation, her only medications were metformin (2000 mg/day) and levothyroxine (50 mcg/day). She had no family history of migraine.

On examination, her vital signs were within normal limits. The physical exam was unremarkable except for a body mass index (BMI) of 28.8, classifying her as overweight [8]. Laboratory studies, including a complete blood count, thyroid-stimulating hormone (TSH), serum glucose, renal function tests, electrolytes, and C-reactive protein, were within normal limits except for mildly elevated TSH with normal thyroxine (T4) levels (Table 1). A repeat blood pregnancy test was negative.

**Table 1:** Laboratory test results of the patient on the day of admission. BUN: blood urea nitrogen; CRP: C-reactive protein; fT3: free triiodothyronine; fT4: free thyroxine; HGB: hemoglobin; Htc; hematocrit; MCV: mean corpuscular volume; MCH: mean corpuscular hemoglobin; MCHC: mean corpuscular hemoglobin concentration; RBC: red blood cell; TSH: thyroid stimulating hormone; WBC: white blood count

	Blood Parameter	Result	Reference Range
Complete Blood Count	WBC, x10^3^/µL	5	4.3-10.3
	RBC, x10^6^/µL	4.54	4.2-5.4
	HGB, g/dL	12.8	12-16
	Htc, %	37.7	36-48
	MCV, fL	83.1	80-99
	MCH, pg	28.3	27.2-33.5
	MCHC, g/dL	34	32-36
	RDW, %	12.8	12-15
	Plt, x10^3^/µL	290	156-373
Basic Metabolic Panel	Glucose, mg/dL	78	74-109
	BUN, mg/dL	15	17-49
	Creatinine, mg/dL	0.45	0.5-0.9
	Sodium, mmol/L	139	136-145
	Potassium, mmol/L	3.84	3.5-5.1
	Chloride, mmol/L	99	98-107
	Calcium, mg/dL	9.18	8.4-10.2
	Magnesium, mg/dL	1.72	1.6-2.6
Acute Phase Reactant	CRP, mg/L	1.11	<5
Thyroid Function Tests	TSH, µIU/mL	6.34	0.27-4.2
	fT3, pg/mL	3.06	2-4.4
	fT4, ng/dL	1.11	0.93-1.7

**Figure 1:**
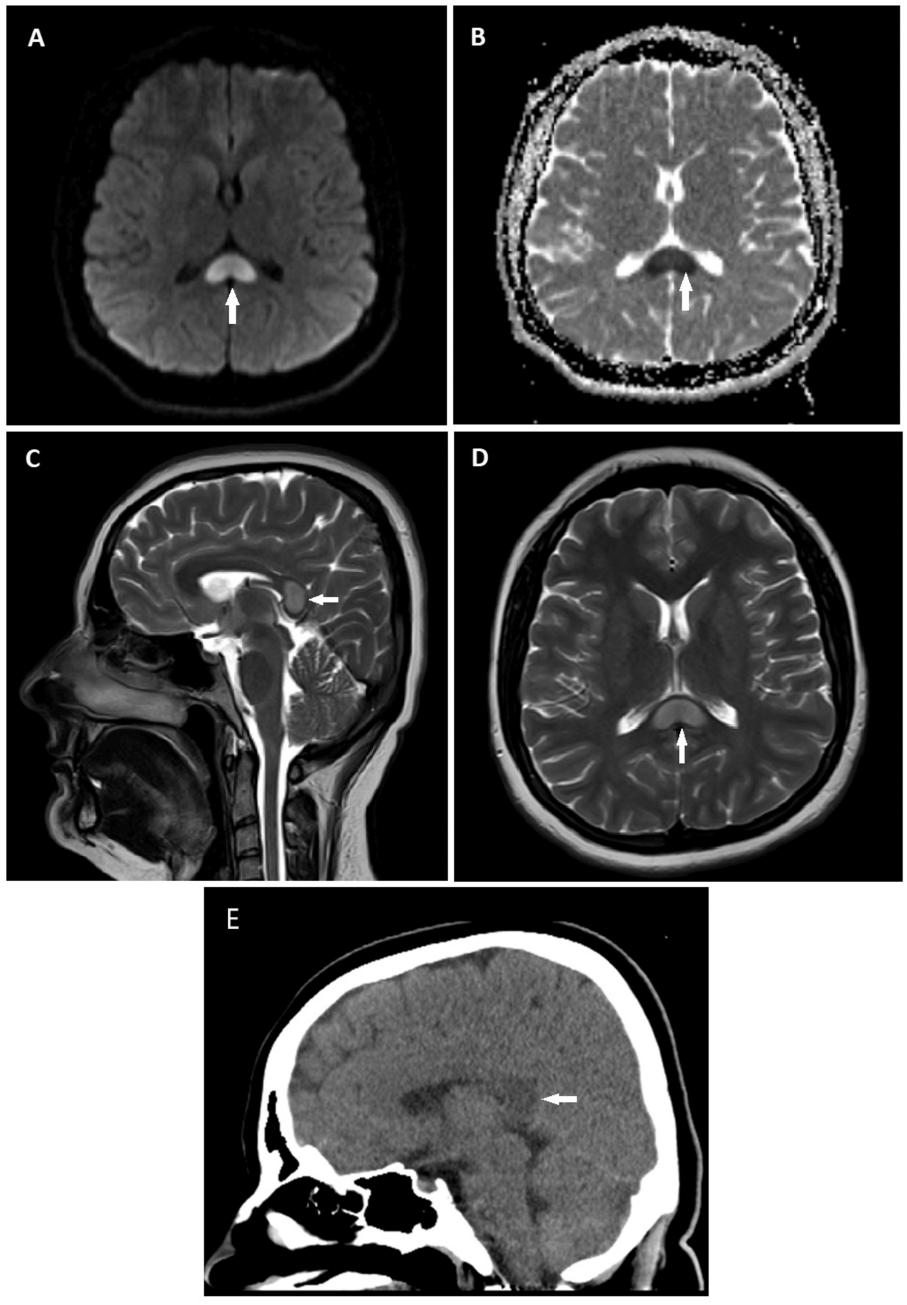
Brain MRI and CT of the case on the day of admission. MRI of the brain shows restricted diffusion in the splenium of the CC with a hyperintense signal on the DWI (A) and a hypointense signal on the apparent diffusion coefficient map (B) (white arrows). The sagittal (C) and axial (D) T2-weighted images reveal the ovoid, well-circumscribed hyperintense lesion within the mid-splenium (white arrows). The sagittal CT image shows a hypodense lesion within the splenium (E) (white arrow). MRI: magnetic resonance imaging; DWI: diffusion-weighted imaging; CT: computed tomography; CC: corpus callosum

Neuroimaging revealed a hypodense lesion in the splenium of the CC on non-contrast CT images of the brain (Figure 1). MRI of the brain with and without contrast showed an oval-shaped, well-circumscribed, T2/FLAIR hyperintense, non-contrast-enhancing focal lesion in the mid-splenium of the CC, which was accompanied by restricted diffusion with a hyperintense signal on DWI and a hypointense signal on the ADC map (Figure 1). Magnetic resonance venography (MRV) of the brain, which was performed to exclude CVT, was unremarkable. Electroencephalography (EEG) was also performed as CC splenium lesions with diffusion restriction were reported in patients with seizures, and it showed normal findings [1].

**Figure 2:**
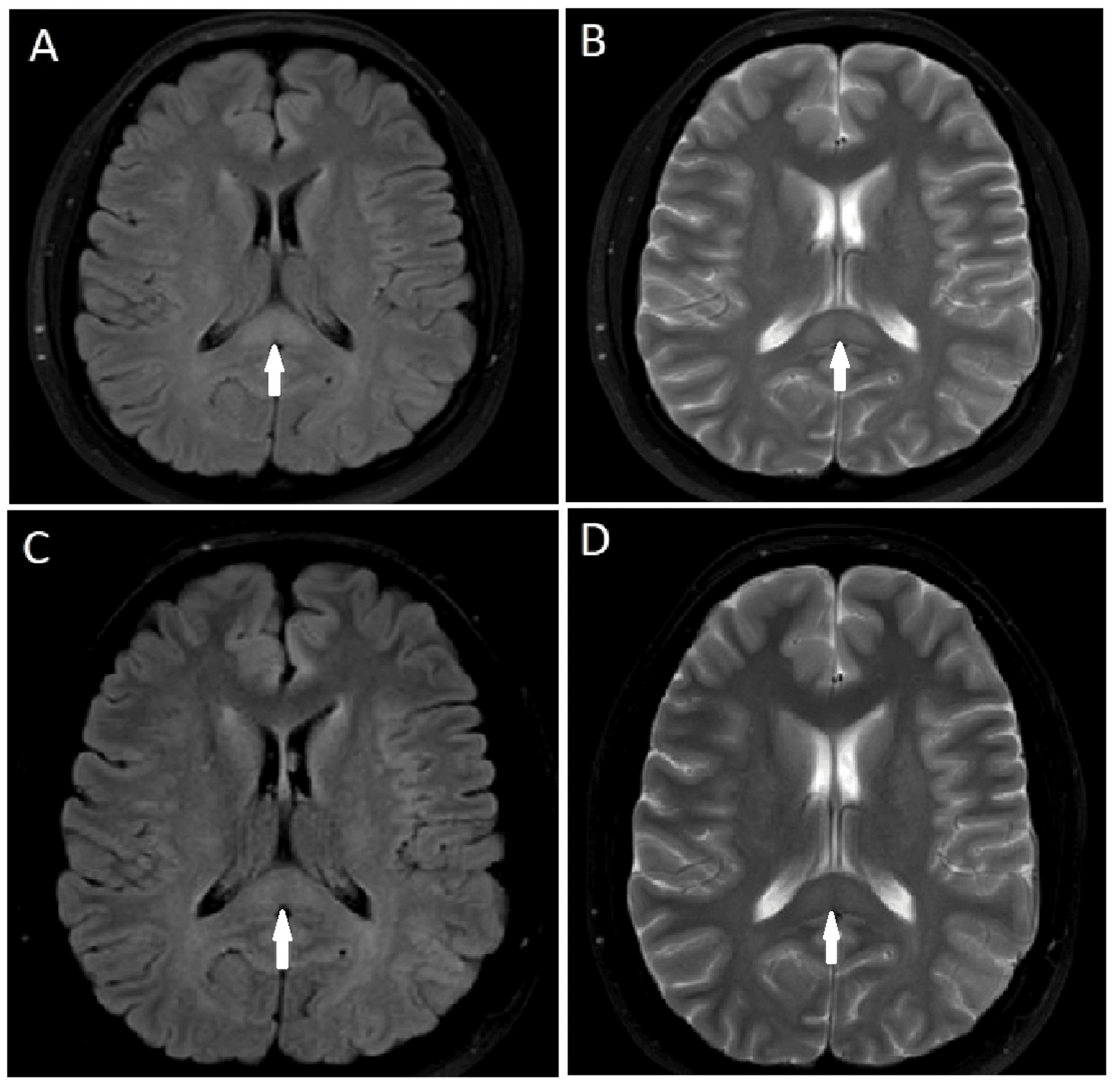
Follow-up brain MRI results. Axial FLAIR (A) and T2-weighted (B) MRI at six weeks demonstrate partial resolution of the lesion (white arrows). Follow-up brain MRI at six months shows near-complete resolution of the lesion in axial FLAIR (A) and T2-weighted (B) images (white arrows). MRI: magnetic resonance imaging; FLAIR: fluid-attenuated inversion recovery

The patient was treated with 1000 mg of intravenous paracetamol infused in 150 mL of normal saline, leading to prompt resolution of her headache. Follow-up brain MRI at six weeks showed complete resolution of the diffusion restriction and partial resolution of the T2 and FLAIR hyperintensity. A repeat MRI at six months demonstrated near-complete resolution of the T2 and FLAIR hyperintensity (Figure 2). Interval follow-up for six months revealed no lasting neurologic deficits.

## Discussion

The CC, particularly the splenium, is highly susceptible to cytokinopathy due to its dense concentration of cytokine, toxin, drug, glutamate, and other excitatory amino acid receptors compared to other brain regions [5,9-11]. While the precise mechanism remains unclear, it is believed that the release of inflammatory cytokines elevates extracellular glutamate levels, leading to water influx into astrocytes and neurons. This, in combination with cytokine storms and oxidative stress, results in cytotoxic edema [5]. Notably, the splenium differs from the rest of the CC in that it receives blood supply from both the anterior and posterior circulations, rather than solely from the internal carotid artery network, and it has a higher density of aquaporin-4 receptors [11]. These factors may explain why the splenium is the most commonly affected region in CLOCCs.

Neuroinflammation plays a critical role in the pathophysiology of migraine, which may explain why CLOCCs are occasionally observed in migraine patients. Glutamate levels have been shown to increase in the blood, cerebrospinal fluid, saliva, occipital cortex, and thalamus both during and between migraine attacks, regardless of headache frequency. Heightened glutamatergic activity in the brain is believed to contribute to increased cerebral excitability and greater susceptibility to cortical spreading depression [9,10]. Several migraine therapies, including magnesium, topiramate, memantine, and ketamine, act on glutamate receptors and have demonstrated efficacy [9]. Structural changes in the CC have also been reported in migraine patients, with more pronounced alterations in those with MwA compared to those without aura [12,13]. As routine neuroimaging is not typically performed in adult or pediatric migraine patients without recent changes in headache patterns, seizures, or focal neurological symptoms, the true incidence of CLOCCs in migraine remains unknown.

Hormonal fluctuations are also a significant contributor to migraine pathophysiology, particularly in women. Many women experience their first migraine attacks around puberty, coinciding with hormonal changes. The majority report increased frequency in the two days preceding menstruation and during the first three days of menses, a pattern largely attributed to the rapid decline in estrogen levels during the late luteal phase. Interestingly, this estrogen drop occurs more rapidly in migraineurs than in controls, even in the absence of a headache, suggesting an underlying neuroendocrine vulnerability [14].

IVF-ET protocols, which are associated with substantial hormonal fluctuations, especially in estrogen levels, may trigger migraine attacks, even in individuals without a prior history. The most widely used regimen, known as the "long protocol," includes two distinct hypoestrogenic phases that may predispose patients to migraine. The first phase occurs during the downregulation of the hypothalamic-pituitary-ovarian axis via gonadotropin-releasing hormone (GnRH) agonists; headache prevalence is reported to be highest during this stage. This is followed by a phase of abrupt estrogen elevation with ovarian hyperstimulation. If embryo transfer fails, a sharp decline in estrogen levels typically occurs two weeks later, marking a second phase of increased headache frequency in non-conceiving patients [6].

In our case, the patient had no prior personal or family history of migraine, suggesting that IVF-ET treatment itself likely triggered the onset of MwA and the associated CLOCC by causing cytokinopathy. Although a lack of headache diary and hormone level data during and after the IVF-ET treatment limits the ability to reach a definitive conclusion, the timing of her most severe episode, 10 days after confirmation of ET failure, supports the “estrogen withdrawal” hypothesis as a migraine trigger [6,14]. The potential role of progesterone levels in IVF-related migraine has not been studied, limiting our ability to assess its contribution in this case. It is unlikely that CLOCC development resulted from direct medication toxicity, as the patient was not actively receiving IVF-ET medications at the time of symptom onset.

Patients with CLOCCs do not typically show symptoms of hemispheric disconnection, such as alien hand syndrome, pseudoneglect, apraxia, agraphia, tactile anomia, visual deficits, and visual neglect, but rather symptoms of the underlying disease [1]. Although case reports suggest favorable short-term outcomes, the long-term effects of CLOCCs remain uncertain. Lower fractional anisotropy values in the CC have been associated with depressive and anxiety disorders in migraine patients, indicating possible under-recognized long-term sequelae [15]. If CLOCCs are incidental acute MRI findings of a chronic process leading to damage in the CC as a result of chronic inflammation in migraine patients, it may be possible that associated subtle chronic symptoms remain unnoticed. Future studies are needed to explore the potential chronic impacts of CLOCCs, especially in those in which the underlying disease entity is chronic. Our patient showed a favorable outcome during her six-month follow-up. However, the lack of long-term neuropsychological follow-up prevents us from concluding that she recovered without any long-term sequelae.

With the rising prevalence of IVF-ET due to delayed childbearing and increasing rates of obesity, the identification and management of neurological side effects, including migraine, are becoming more clinically relevant [16,17]. Patients may underreport headaches, assuming them to be benign side effects of treatment. Although our patient was eligible because of the negative blood pregnancy test performed 10 days ago, she was not on any migraine-specific medical care at the time she developed CLOCC. Physicians should be vigilant about the possibility of underreported migraine symptoms in individuals undergoing IVF-ET and should consider implementing routine headache screening. Integrating targeted treatment strategies, such as migraine-specific abortive or preventive therapies prior to pregnancy, or estrogen "add-back" therapy during hypoestrogenic phases, may improve patients' quality of life and reduce the risk of complications [6].

## Conclusions

IVF-ET has become an increasingly common fertility treatment, driven by trends such as delayed childbearing and rising obesity rates. However, IVF-ET can provoke migraine headaches and related complications, including CLOCCs, likely due to the pronounced hormonal fluctuations involved. Patients may underestimate the significance of these headaches, attributing them to routine side effects of fertility treatment, as in our case. It is essential to monitor individuals undergoing IVF-ET treatment for headache symptoms and to implement appropriate management strategies. Doing so can improve daily quality of life and help prevent both acute and long-term neurological complications associated with migraine.
